# Supplementary material for: A high-resolution mRNA expression time course of embryonic development in zebrafish
Source: eLife. 2017 Nov 16;6:e30860. doi: 10.7554/eLife.30860 (PMC5690287; doi:10.7554/eLife.30860)
Supplement: Supplementary file 6. [file elife-30860-supp6.zip › biolayout-clusters-files/Cluster223-genes.html]

Cluster223


# Cluster223: Genes

| Ensembl ID | Gene Name | Chr | Start | End | Biotype |
| --- | --- | --- | --- | --- | --- |
| ENSDARG00000056829 | CR559945.1 | 13 | 11633960 | 11667740 | protein\_coding |
| ENSDARG00000010280 | clasp1a | 9 | 38354081 | 38482020 | protein\_coding |
| ENSDARG00000062868 | eea1 | 18 | 15872770 | 15913617 | protein\_coding |
| ENSDARG00000027322 | lin7c | 7 | 31631184 | 31650466 | protein\_coding |
| ENSDARG00000043905 | rnf14 | 21 | 41826233 | 41848869 | protein\_coding |
| ENSDARG00000036793 | ttll5 | 17 | 51851747 | 51983272 | protein\_coding |
